# Pulmonary Aspergillosis in a Previously Healthy 13-Year-Old Boy

**DOI:** 10.1155/2016/4575942

**Published:** 2016-02-25

**Authors:** Jonathan H. Rayment, Indra Narang

**Affiliations:** Division of Respiratory Medicine, The Hospital for Sick Children, Toronto, ON, Canada

## Abstract

Chronic granulomatous disease (CGD) is a rare, polygenic primary immunodeficiency. In this case report, we describe a previously healthy 13-year-old boy who presented with multifocal pulmonary aspergillosis and was subsequently diagnosed with an autosomal recessive form of chronic granulomatous disease. CGD has a variable natural history and age of presentation and should be considered when investigating a patient with recurrent or severe infections with catalase-positive organisms.

## 1. Case Report

A 12-year-old boy presented to the Paediatric Emergency Department with a three-month history of daily fever, migratory arthritis, angular cheilitis, a reported 5 kg weight loss, and malaise. Past medical history was unremarkable other than immigration from Pakistan to Canada at the age of 7. Family history was significant for parental consanguinity (first cousins), but both parents and an older brother (17 years) and sister (19 years) were reported as being well.

He was admitted to hospital for a diagnostic evaluation and was found to have systemic inflammation (elevated c-reactive protein, erythrocyte sedimentation rate, ferritin, sCD-163, and sCD-25) concerning macrophage activation syndrome (MAS). He underwent an extensive workup for potential secondary causes of this inflammation. Bacterial blood culture, EBV/CMV,* Toxoplasma gondii*, parvovirus B19 and* Legionella* serologies, respiratory virus multiplex PCR, and TB skin test were negative. An abdominal ultrasound and bone marrow aspirate showed no evidence of malignancy. An autoantibody panel was sent and was positive only for anti-nuclear antibody (1 : 160, speckled pattern). A chest X-ray was performed and was normal.

At this time, a clinical diagnosis of systemic onset juvenile idiopathic arthritis was made. Treatment with oral prednisone (60 mg orally, once daily) was initiated on day 3 of admission, which resulted in rapid resolution of the fevers, arthritis, and cheilitis. He was discharged home on day 6 of admission with follow-up planned with Rheumatology.

**Figure 1:**
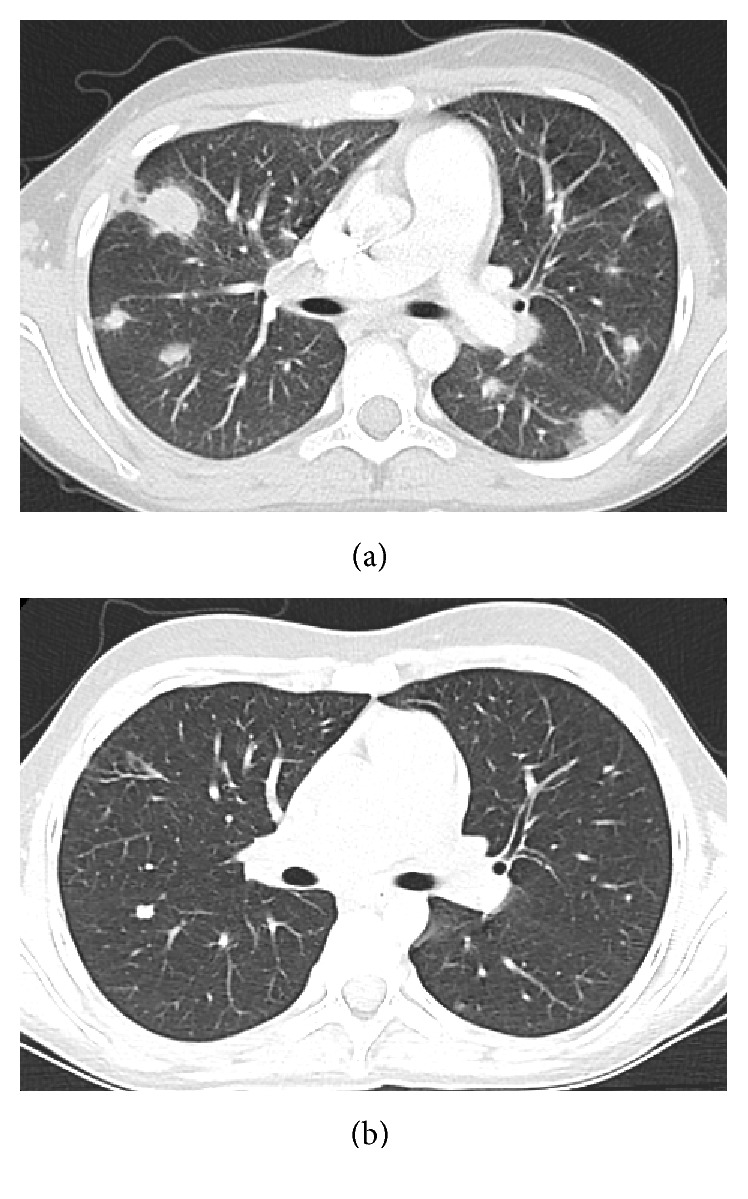


He presented to the Emergency Department 3 weeks later with a three-day history of fever, cough, and pleuritic chest pain. Chest X-ray revealed bilateral, multifocal pulmonary nodules and chest CT confirmed the presence of bilateral, predominantly apical homogeneous nodules (5–20 mm in diameter) and hilar lymphadenopathy (Figure 1(a)).

The main differential diagnosis of this child's pulmonary nodules was infectious. A bronchoalveolar lavage revealed the presence of* Aspergillus fumigatus* and treatment with voriconazole was subsequently initiated.

Despite the recent initiation of corticosteroids, we were suspicious of a primary immune deficiency in this child with no other apparent risk factors for* Aspergillus* infection [1]. On further questioning, the patient had no personal history of sinopulmonary, skin, or abdominal infections. There was no history of thrush, severe viral infections, or warts and his vaccinations had been administered with no adverse effects. However, his 17-year-old brother did have a history of one episode of suppurative cervical adenitis and a perianal abscess within the past five years, both of which were treated medically without complication.

An immunological workup was pursued and the patient was found to have normal cellular and humoral immune function, but he had a markedly decreased neutrophil oxidative burst index (NOBI) (1.26, normal range: 32–300), which is diagnostic of chronic granulomatous disease (CGD). Subsequently, on genetic analysis, both the patient and his older brother were found to be homozygous for a mutation in the* gp47* ^*phox*^ gene (c.75_76delGT), which is known to be causative for autosomal recessive CGD. His parents and an older sister were all found to be heterozygous for the same mutation.

Over the ensuing 12 months, the patient was treated with oral voriconazole and prophylactic oral cotrimoxazole and the lung lesions improved considerably on chest CT (Figure 1(b)). There have been no further significant pulmonary or extrapulmonary infections.

## 2. Discussion

CGD is a rare (1 : 250,000) polygenic primary immunodeficiency that results from a defective NADPH oxidase protein complex. NADPH oxidase is an enzyme complex that generates hydrogen peroxide (H_2_O_2_) and reactive oxygen species (ROS) in the phagosome, which are critical to the oxidative killing of bacteria [2]. Recurrent infections are the primary cause of mortality in these patients. Infections can involve any organ, but pulmonary infections with catalase-positive organisms (*Aspergillus* spp.,* Staphylococcus *spp.,* Burkholderia cepacia*,* Nocardia* spp., etc.) are the most common [3].

Catalase is a bacterial enzyme that detoxifies H_2_O_2_ (which is both endogenously produced by the bacteria and exogenously produced by phagosomal NADPH oxidase) by converting it to water and oxygen. As such, the presence of bacterial catalase is protective against oxidative killing [2]. It is thus theorized that since patients with CGD have significantly decreased phagosomal H_2_O_2_ available to participate in oxidative killing, catalase-positive organisms are more virulent in these hosts.

The genes associated with CGD encode the different subunits in the NADPH oxidase complex. The most commonly affected gene (in approximately 75% of cases) is* gp91* ^*phox*^, which is encoded on the X chromosome, hence the predominantly X-linked inheritance pattern of this condition. Mutations in four other somatically encoded subunits have also been identified and account for the 25% of CGD that is recessively inherited [3].

The natural history of CGD is variable and ranges from overwhelming infections with early childhood death to a relatively benign course with survival into later adulthood. X-linked CGD tends to manifest a more severe phenotype than the autosomal recessive disease [3]. This genotype-phenotype association has been shown to be linked directly to residual* in vitro *NAPDH oxidase enzymatic activity. Mutations in the X-encoded* gp91* ^*phox*^ gene result in significantly less residual function than mutations in the somatically encoded genes [4].

Pulmonary manifestations of CGD are primarily infectious, with catalase-positive organisms. However, sterile pulmonary granulomatous reactions are also common in these patients [5]. Pulmonary inflammatory responses can be severe, especially after exposure to a high inoculum of* Aspergillus*, and have been termed CGD “mulch pneumonitis.” Mulch pneumonitis can present with acute respiratory distress syndrome and is potentially life-threatening. It can, however, be very responsive to a combination of antifungal and corticosteroid therapy [6].

The mainstay of treatment of CGD is antimicrobial prophylaxis. Prophylactic cotrimoxazole [7] and itraconazole [8] have been shown to decrease the number of bacterial and fungal infections in these patients. Interferon gamma has also been shown to reduce the frequency of severe infections [9]. Finally, bone marrow transplant can be considered, especially in patients with mutations that are predicted to cause severe disease [4].

In our case, an adolescent boy presented with pulmonary aspergillosis and was subsequently diagnosed with CGD. This rare, polygenic primary immunodeficiency has a variable natural history and age of diagnosis. Pulmonary manifestations of CGD are common, and they should be considered as an underlying diagnosis when investigating a patient with severe or recurrent pulmonary infections with catalase-positive organisms such as* Aspergillus*.

## Pretest

1. What are the pulmonary manifestations of chronic granulomatous disease?
2. Why are CGD patients susceptible to catalase-positive microorganisms?

## Learning Objectives

1. Recognise the pulmonary manifestations of chronic granulomatous disease.
2. Recognise the organisms to which CGD patients are particularly susceptible.

## Posttest

1. The pulmonary manifestations of CGD are mostly infectious, with pneumonia, empyema, and bronchiectasis. However, sterile granulomas are also seen. ARDS-like inflammatory reactions can also be seen in patients who inhale large inoculums of* Aspergillus* spores (“mulch pneumonitis”).
2. Due to the genetic defect in the NADPH oxidase complex, patients with chronic granulomatous disease are unable to mount an effective phagosomal oxidative burst. Minimal residual NADPH activity and endogenous (bacterially produced) ROS may be sufficient to kill some organisms, but catalase-positive organisms are able to detoxify this low level of H_2_O_2_. As such, CGD patients are particularly susceptible to infections by catalase-positive microorganisms.
